# Supplementary material for: Design, Fabrication and Characterisation of Multi-Parameter Optical Sensors Dedicated to E-Skin Applications
Source: Sensors (Basel). 2022 Dec 23;23(1):114. doi: 10.3390/s23010114 (PMC9824189; doi:10.3390/s23010114)
Supplement: Supplementary file 1 [file sensors-23-00114-s001.zip › Supplementary-Information.pdf]

Supplementary Information: Design, fabrication and characterisation of multi-parameter optical sensors dedicated to e-skin applications

Lionel Fliegans, Joseph Troughton, Valentin Divay, Sylvain Blayac, Marc Ramuz

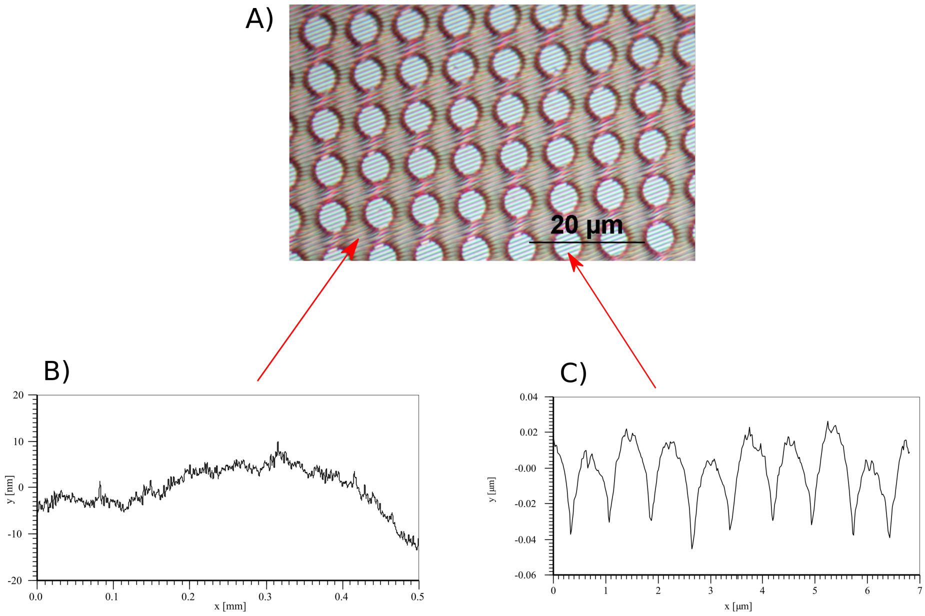

Figure S1: A) Optical microscope image of high resolution photoresist pattern. B) Line profile of S1813 photoresist. C) AFM picture of the grating coupler PDMS topographies after molding.

A)

B)

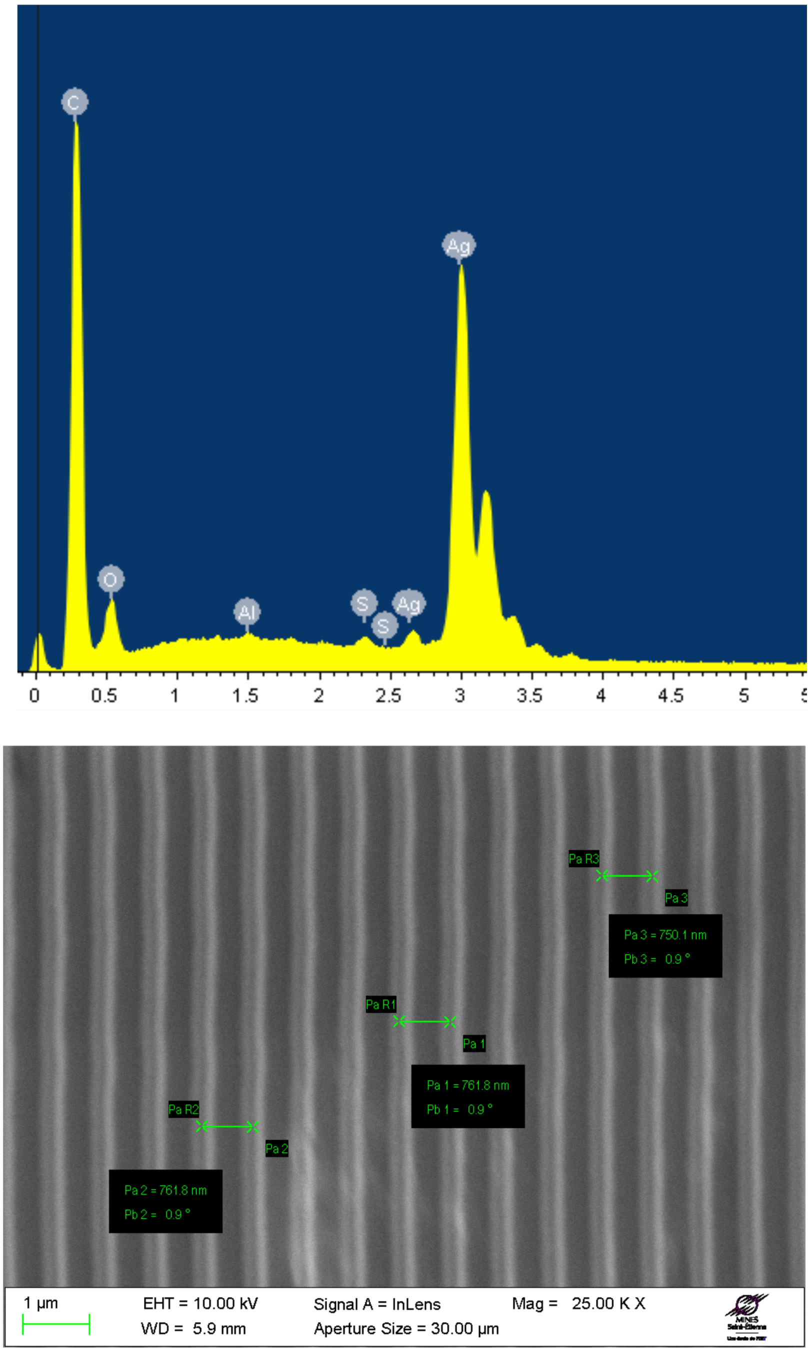

Figure S2: A) EDX spectrum analysis on metal layer. B) SEM scanning of the grating silver layer.

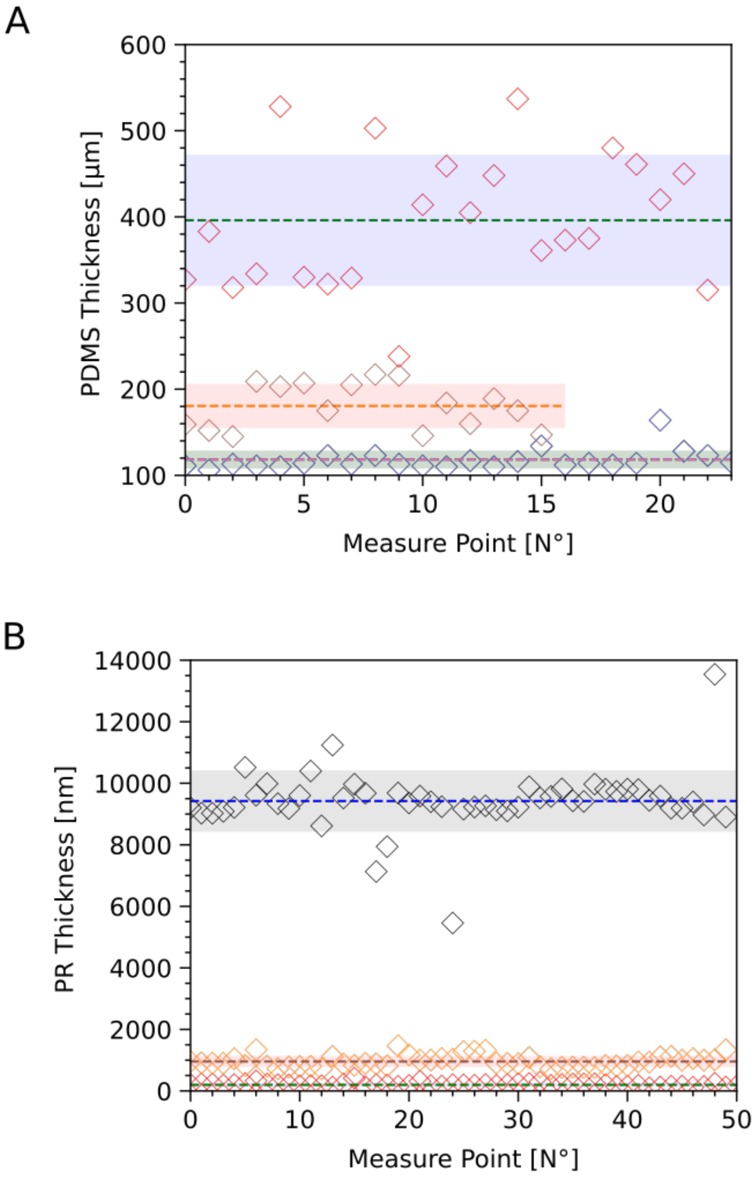

Figure S3: A) The thickness (D) of the waveguides for different spin-coating speeds of PDMS. B) The thickness (d) of the grating zone for different spin-coating speeds of photoresist.

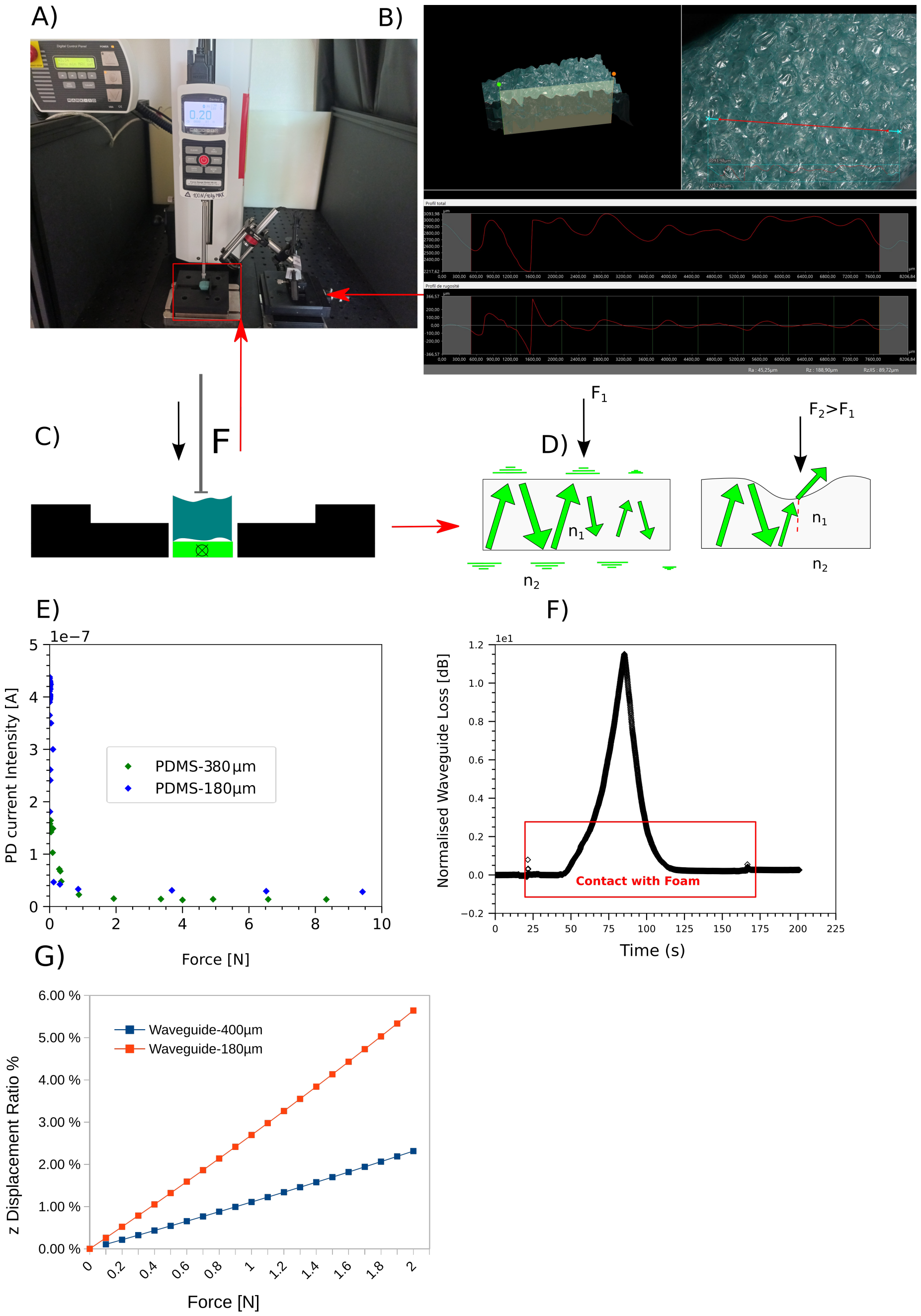

Figure S4: A) Compression force test bench. B) Numerial microscope image and surface roughness parameters of PE foam. C) Schematic representation of measurement setup see from left side. D) Schematic representation with the two attenuation behaviours: Loss by evanescent field and loss by mechanical deformation. E) Waveguide response under compression force without foam. F) Normalized waveguide loss versus time for one cycle of applied force: Before and after contact with the foam. G) Finite element analysis (Comsol) of the geometric deformation of a waveguide with two thicknesses (400 μm and 180 μm) with applied compression force.

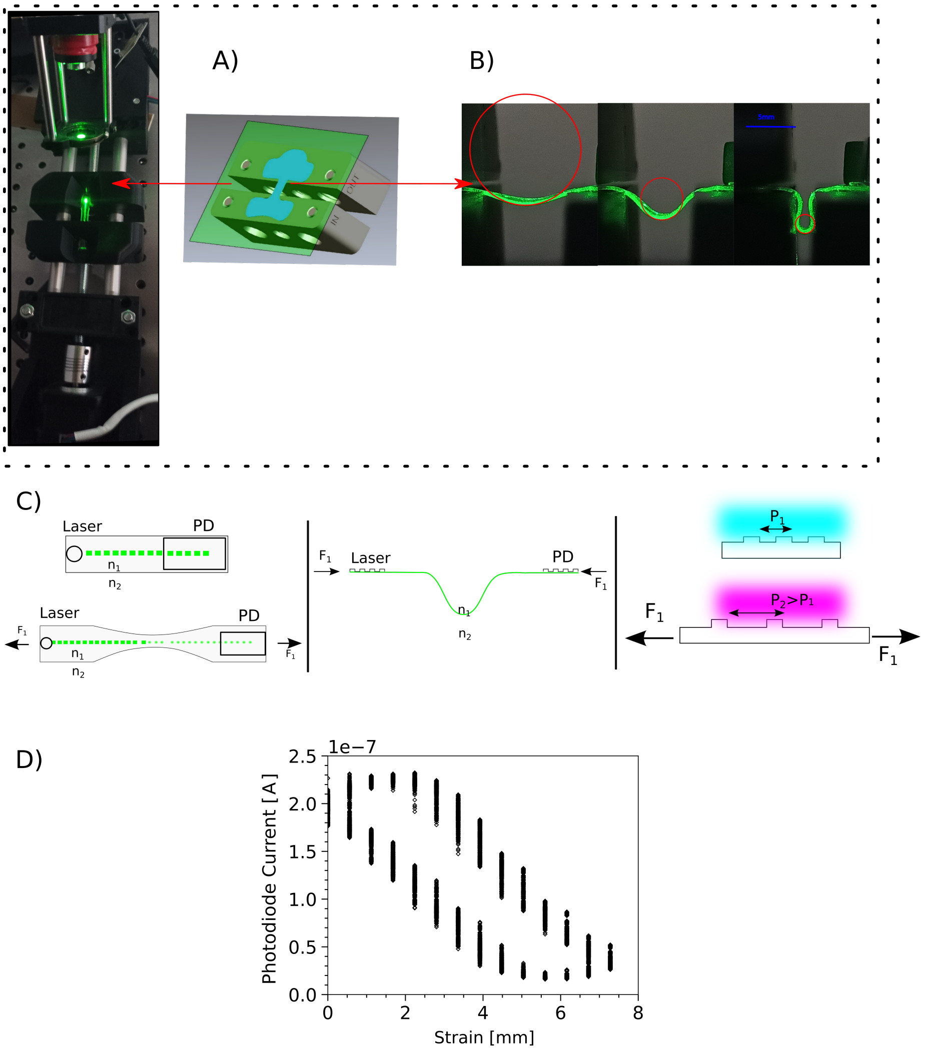

Figure S5: A) Strain test bench. B) Measurement of bending radius. C) Schematic representation of the waveguide under different types of mechanical stress: Strain sensor, Bending sensor, Color sensor. D) Measured photo-current from the device over 100 cycles at a rate of 0.8 mm/s.

Normalised frequency	$D(\mu m)/\lambda(\mu m)$	Stretch sensor loss (dB)	Compression force sensor loss (dB)	Bending sensor loss (dB)
$V \approx 2124$	180/0.532	$0.1 \times \epsilon$	$9.75 \log(6.59F + 1.0)$	$121.55 \exp(-2.70R_b)$
$V \approx 2513$	180/0.450	NaN	$8.37 \log(3.10F + 0.98)$	NaN
$V \approx 4486$	400/0.532	NaN	$9.0 \log(1.39F + 0.98)$	NaN
$V \approx 5278$	400/0.450	NaN	$10.95 \log(0.78F + 0.98)$	NaN

Table S1: Summary of the different losses under different types of strain.
